# The National Institute on Alcohol Abuse and Alcoholism: Past Accomplishments and Future Goals

**Published:** 1995

**Authors:** Enoch Gordis

**Affiliations:** Enoch Gordis, M.D., is director of NIAAA

**Keywords:** government agency, AOD abuse, AOD dependence, research funding

## Abstract

Thanks largely to support from the National Institute on Alcohol Abuse and Alcoholism, alcoholism researchers have made significant strides in understanding the causes, prevention, and treatment of alcoholism and its consequences. Ongoing research will extend this knowledge.

The creation in 1970 of the National Institute on Alcohol Abuse and Alcoholism (NIAAA) led to major national interest in research toward solving the problems of alcohol abuse and alcoholism. Although several distinguished scientists had made important contributions to the field prior to that time, funding had been inconsistent, and multidisciplinary research on alcoholism was almost nonexistent (Lieber 1989). With a portfolio that includes internationally respected investigators in a broad range of biomedical and psychosocial disciplines, NIAAA today provides leadership and financial support for approximately 90 percent of all alcohol-related research in the United States. This article reviews some of the major advances in alcohol research in which NIAAA has participated and looks forward to the achievements of the future.

## Alcoholism Is a Disease

An essential impetus to the development of alcoholism research was the acceptance of alcoholism as a medical disorder. This concept has evolved progressively and with considerable controversy over the past 200 years (Jaffe 1993). Competing views have included the notion of alcoholism as a symptom of psychological maladjustment or as the arbitrarily delineated upper end of a continuum of drinking (Keller 1990).

The disease concept defines alcoholism as an independent disorder characterized by a craving for alcohol—a dependence, or addiction. As such, alcoholism is distinguished from drinking that is merely heavy, problematic, ill advised, or socially unacceptable. True, many alcohol-related problems result from misuse of alcohol by persons who are not alcoholic. Nevertheless, the disease concept has sharpened the focus of alcohol research and has helped remove the stigma from a chronic disorder that is no more inherently immoral than diabetes or heart disease.

## What Is Alcoholism and What Accounts for It?

Alcoholism is characterized by abnormal alcohol-seeking behavior and impaired control over drinking. Significant phenomena related to the development of alcoholism include reinforcement, tolerance, and both physical and psychological dependence. These phenomena contribute to the rewarding effects of alcohol, the persistence of drinking, and the ultimate development of alcoholism in people who are vulnerable to the disease for underlying genetic/environmental reasons.

The actions of alcohol on certain brain centers may lead to sensations perceived as rewarding, or pleasurable. The process by which a person learns to repeat rewarding behavior is called positive reinforcement. This process encourages the persistence of drinking in persons who are vulnerable for underlying genetic or psychosocial reasons (see below) (Wise and Rompre 1989).

In contrast, the process by which a person learns to repeat behavior that reduces unpleasant sensations is called negative reinforcement. For example, alcohol may help relieve stress in people who are prone to stress as a result of either genetics or learned behavior (Sher and Levenson 1982).

Tolerance means that more of a drug is required to achieve a given effect. This may lead to increased alcohol consumption and increased damaging effects from use of alcohol (Harris and Buck 1990).

The hallmark of physical dependence is the occurrence of a withdrawal syndrome when alcohol consumption is discontinued after a period of heavy drinking. Alcohol withdrawal symptoms include anxiety; agitation; increased blood pressure; and, in extreme cases, seizures. These symptoms may persist for several days.

Psychological dependence is characterized by craving, a mental state involving a strong need for alcohol that can occur even in the absence of physical dependence (Harris and Buck 1990).

The key question in alcoholism research is, Why do some people exhibit a pathological appetite for alcohol whereas others do not? Studies using experimental animals have revealed that the actions of alcohol that cause intoxication, reinforce drinking behavior, and lead to addiction are based principally in the brain. NIAAA has dedicated a large proportion of its research effort to this area.

Researchers once believed that alcohol influenced brain function through some general disturbance of nerve cell membranes. This view has been modified by the growing recognition that alcohol preferentially affects specific molecular components of nerve cell membranes. These molecular components include receptor proteins crucial for communication among nerve cells.^1^ In one clear demonstration of a specific alcohol-receptor interaction, NIAAA researchers revealed that alcohol can alter the function of the N-methyl-D-aspartate (NMDA) receptor, one of three subtypes of receptor that respond to the excitatory neurotransmitter glutamate. Significantly, this effect occurs at alcohol concentrations present in the brain after only one drink (Lovinger et al. 1989). The effect of alcohol on this receptor may contribute to the phenomena of intoxication and withdrawal (Hoffman et al. 1989; for a further discussion, see the article by Linnoila and colleagues, pp. 60–70).

NIAAA investigators have found additional associations between alcohol and specific neurotransmitters whose actions might help mediate alcohol reinforcement, intoxication, and dependence (Linnoila 1989; Harris and Buck 1990). For example, a neurotransmitter that appears to enhance long-term memory also maintains tolerance to alcohol after alcohol consumption has ceased. This suggests that the development of tolerance may be related to the processes of learning and memory (Hoffman et al. 1978; Tabakoff and Hoffman 1988).

Researchers are seeking additional associations between alcohol and specific neurotransmitters. However, the complex mental processes that govern drinking behavior are unlikely to be carried out by a small number of independent neurotransmitter-receptor interactions. The existence within the brain of multiple intersecting pathways of nerve cell communication has generated a new emphasis on the study of neural networks—integrated brain circuits that modulate one another’s activity. Alcohol may influence the activity of a given communication pathway by interacting with receptors on nerve cells within the pathway or on nerve cells that modulate these circuits (Dworkin and Smith 1991).

## Why Are Some People More Vulnerable to Alcohol’s Effects?

A combination of genetic and environmental factors influence vulnerability to alcoholism.

An important benchmark in the history of alcoholism research was the demonstration that a significant portion of the susceptibility to alcoholism is inherited. Understanding the genetic contribution to the development of alcoholism helps clarify the nature of the disorder and provides the basis for early recognition, treatment, and prevention efforts. From its inception, NIAAA has supported studies in alcoholism genetics utilizing a range of techniques from the investigation of family pedigrees to the latest methods of molecular analysis.

### Genetic Factors

#### Population-Based Studies

Genetic alcoholism research began with the observation that alcoholism “runs in families.” To determine the extent to which this relationship is affected by biological inheritance, NIAAA has contributed to a variety of population genetics studies over the past 20 years.

Adoption studies indicate that children of alcoholics raised in nondrinking homes continue to face an increased risk of alcoholism (Cloninger et al. 1981; Goodwin et al. 1973). Other studies have shown that the identical twin of an alcoholic has a 60-percent probability of also becoming alcoholic, whereas the fraternal twin of an alcoholic has only a 36-percent chance of developing alcoholism (reviewed in Pickens and Svikis 1991). These findings suggest that genetic inheritance is a major contributing factor behind the development of alcoholism.

#### Trait Markers

Certain abnormalities of biochemical function may be inherited along with a predisposition to developing alcoholism. These abnormalities may serve as trait markers for identifying persons at risk for the disease. For example, alcoholics appear to demonstrate differences in certain components of brain electrical activity compared with nonalcoholics (Porjesz and Begleiter 1979; Begleiter et al. 1980; Pfefferbaum et al. 1979). Sons of alcoholic fathers display the same differences compared with sons of nonalcoholic fathers in response to a visual stimulus (Begleiter et al. 1984).

The activity of a brain enzyme involved in neurotransmitter metabolism is another potential trait marker. This enzyme, monoamine oxidase (MAO), also is found in blood platelets, where its activity can easily be determined. The finding that MAO activities are controlled genetically has led to a hypothesis that diminished MAO levels observed in alcoholics may be a biochemical marker for genetic susceptibility to alcoholism (Buchsbaum et al. 1976).

### Molecular Genetics Studies

#### Cooperative Agreement on Genetics of Alcoholism (COGA)

A range of genetic research tools is being brought to bear in a current multisite, multidisciplinary genetic study known as COGA. In partnership with NIAAA, COGA will examine approximately 3,000 subjects from several hundred families with alcoholic pedigrees. The long-term objective of this research is to pinpoint the chromosomal location of genes that influence susceptibility to alcoholism. State-of-the art technology is being used to screen the entire length of each chromosome for genes that may be linked to alcoholism. This search relies on the existence of microsatellite repeat markers—short, repeating chromosomal segments that differ from one person to another. To assist the search, scientists are adapting techniques developed for studying single-gene diseases to the more complex disease of alcoholism. Results of COGA will help identify persons at high risk for alcoholism and also will aid in the development of new treatments for alcohol-related problems.

#### Genetic Animal Models

NIAAA has long played an important role in developing animal models for alcohol research, particularly in the area of genetics. Many rat and mouse strains have been selectively bred for sensitivity to various effects of alcohol. The development of such strains demonstrates that the selected trait is, to some extent, genetically determined. In addition, researchers use selectively bred animal lines to investigate the biological mechanisms of alcohol’s effects (Crabbe and Phillips 1990).

Genetic animal models display many characteristics of human alcoholics. Li and colleagues (1981) developed an alcohol-preferring (P) line and an alcohol-nonpreferring (NP) line of rats that have, respectively, a high and low preference for alcohol. The P rats develop physical dependence with long-term exposure, exhibiting signs of withdrawal when alcohol is discontinued (Crabbe and Phillips 1990). Moreover, alcohol tolerance develops more rapidly and persists longer in P than in NP rats (Li et al. 1987).

Studies in these and other selectively bred strains have demonstrated that tolerance and dependence are probably not controlled by a single mechanism, because it is possible to alter alcohol tolerance without affecting alcohol dependence (Tabakoff and Ritzmann 1977). Moreover, evidence from both human and animal studies suggests that tolerance and dependence are controlled not only by different genes but by different sets of genes.

As these findings illustrate, susceptibility to complex behavioral disorders such as alcoholism is not likely to be determined by a single gene. Therefore, NIAAA-supported investigators are using a new technique in animal studies called quantitative trait locus (QTL) mapping. This technique makes it possible to define the contribution of a single gene and its variations to complex behaviors that are determined by the interaction of many genes. Results of QTL mapping in animals can then be used to guide the search for similar genes in the human genome.

### Environmental Factors

Sociocultural factors provide a framework for beliefs and attitudes about alcohol that encourage drinking and may lead to addiction. NIAAA has sponsored studies on risk factors and determinants of drinking in the home and in society at large as well as those based on ethnicity and gender.

In the past decade, NIAAA-sponsored researchers have investigated mental processes involved in drinking, including decisionmaking, learning, and expectancies. For example, expectancies, particularly beliefs about the positive effects of alcohol, have been found to predict drinking behavior. Findings also indicate that children have positive expectations about alcohol’s effects as early as age 8 (Miller et al. 1990).

The role of both genetic and environmental factors in alcohol-related behavior can be determined more precisely when studied together. Moreover, in addition to influencing drinking behavior directly, environmental factors may affect the actual structure and function of the nervous system. Thus, the distinction between behavioral and biomedical science is rapidly disappearing. The interplay among genetics, neuroscience, and behavior will form the basis for promising new approaches to prevention and treatment.

## Alcohol’s Medical Effects

Research in the past 25 years has brought about an increasing awareness of the multiplicity of alcohol’s medical effects and the mechanisms by which these effects occur. Some of these effects include alcoholic cirrhosis; cancer; immune defects; cognitive impairment; fetal alcohol syndrome (FAS); and alcohol-related trauma, including accidents, traffic crashes, and personal violence. Some prevention efforts aimed at alcohol-involved driving also are discussed below.

### Alcoholic Cirrhosis and Other Organ Damage

Alcoholic liver disease is one of the most serious medical consequences of chronic alcohol use. Moreover, chronic, excessive alcohol use is the single most important cause of illness and death from liver disease in the United States (Grant et al. 1988). The most advanced form of alcoholic liver injury is alcoholic cirrhosis. This condition is marked by progressive development of scar tissue that chokes off blood vessels and distorts the liver’s internal structure. Although alcoholic cirrhosis can stabilize with abstinence, the disease is often progressive and fatal (NIAAA 1993*a*).

Before the 1970’s, scientists generally attributed alcoholic cirrhosis to the nutritional deficiencies common among heavy drinkers. Research subsequently proved that alcohol is toxic to the liver, even when nutrition is adequate (Lieber 1989).

NIAAA-sponsored research suggests that alcohol is directly toxic to other tissues as well, including bone marrow (Ballard 1993), testicular cells, and both heart and skeletal muscle (Rubin 1989). Research emphasis has shifted from documenting individual organ effects to determining mechanisms of injury at the cellular level.

These and other discoveries suggest that the bewildering diversity of alcohol’s medical effects may be explained by a relatively small number of molecular mechanisms (Rubin 1993). Elucidating these mechanisms may provide the basis for successful treatments for the medical effects of alcoholism.

### Cognitive Impairment

The incidence of alcohol-related organic brain disease accounts for approximately 10 percent of adult dementia cases in the United States (Oscar Berman 1990). Alcoholic dementia is characterized by a global loss of intellectual abilities. Wernicke’s disease is an alcohol-related brain degeneration that produces general confusion, abnormal gaze and gait, loss of muscle coordination, and incoherent speech. This disorder is associated with a deficiency of the vitamin thiamine. Abstinence and vitamin supplementation can reverse this disorder. About 80 percent of alcoholic patients who recover from Wernicke’s disease are left with a residual severe memory disturbance known as Korsakoff’s syndrome, characterized by a permanent state of cognitive dysfunction and an inability to remember recent events or to learn new information (Oscar Berman 1990).

NIAAA is a pioneer in applying sophisticated techniques to produce images of the living human brain for examining brain structure and function in alcoholic organic brain disease. The long-term goals of these studies include predicting alcohol-induced brain damage in time to reverse it and providing rapid feedback on the effectiveness of medications in individual patients (Tabakoff 1989).

### Fetal Alcohol Syndrome

A research milestone of the 1970’s was the identification of FAS, a pattern of birth defects in children of heavy-drinking mothers. FAS is the leading known preventable cause of mental impairment, affecting 1 to 3 infants per 1,000 live births in the United States (NIAAA 1991). The adverse effects of prenatal alcohol exposure exist along a continuum, with complete FAS at one end of the spectrum and incomplete features of FAS, including more subtle cognitive-behavioral deficits, at the other (NIAAA 1991).

NIAAA-sponsored researchers have conducted studies using experimental animals to answer many important questions about FAS. One of the first issues that researchers addressed was whether alcohol itself is responsible for FAS. Alcoholic women frequently smoke tobacco, have poor health, and are malnourished, any of which factors can cause birth defects. Two different laboratories reported that alcohol administration to pregnant mice resulted in birth defects similar to those of FAS (Chernoff 1977; Randall et al. 1977), suggesting that alcohol itself caused the defects.

Additional animal research has demonstrated that critical periods occur during pregnancy in which different organ systems are most susceptible to damage. The specific type of birth defect produced appears to depend on which organ systems are undergoing development at the time of alcohol exposure (Webster 1989).

The existence of critical periods for fetal damage suggests the importance of the mother’s drinking patterns in producing fetal injury. Animal research has shown that peak blood alcohol level, rather than total amount of alcohol consumed, may represent the critical dose of alcohol above which an adverse effect will occur (West et al. 1990). This finding implies that drinking pattern is critical: Rapid consumption of alcohol produces a higher blood alcohol level than does slower consumption of the same amount over a long period. These results suggest the importance for researchers and clinicians of asking women about their drinking patterns in addition to the total number of drinks they consume.

In 1974 NIAAA initiated the first in a series of studies examining the effects of light to moderate alcohol consumption by pregnant women. The offspring of women who drank moderately during pregnancy were found to be smaller in weight, height, and head circumference (Day and Richardson 1994). The effects of prenatal exposure to low levels of alcohol on intellectual development fall along a continuum; the decrement in intellectual function may persist until at least age 7 (Streissguth et al. 1990). Researchers are attempting to determine the threshold alcohol concentration below which these effects might not occur. An average of seven drinks per week may be the threshold for some neurological and behavioral symptoms (Jacobson and Jacobson 1994).

NIAAA-supported research led to the issuance in 1981 of the Surgeon General’s health advisory on risks of drinking during pregnancy.

### Cancer

Alcohol is strongly associated with cancers of the esophagus, pharynx, and mouth; a more controversial association links alcohol with liver, breast, and colorectal cancers. Together these cancers kill more than 125,000 people annually in the United States (NIAAA 1993*b*).

Researchers have identified an enzyme system in the liver that metabolizes alcohol and other ingested chemicals. The normal function of this system is to detoxify harmful chemicals. However, in some cases the effect may be the reverse, increasing the transformation of nontoxic environmental chemicals into substances that may cause cancer (Lieber 1992).

### Alcohol-Related Trauma

Alcohol-induced impairment is associated with a variety of serious and fatal injuries, including those incurred in aviation, boating, cycling, and motor vehicle crashes; drownings; fires; and household accidents. In addition, incidents of personal violence—including domestic violence, suicides, and homicides—are often associated with alcohol use. An important source of alcohol-related trauma is drinking and driving. Of the 40,115 traffic fatalities that occurred in 1993, 17,461 were alcohol related (U.S. Department of Transportation [USDOT] 1994). Statistics indicate that almost two in five Americans will be involved in an alcohol-related crash at some time in their lives (USDOT 1994). NIAAA research has provided the basis for prevention efforts, as described below.

### Immune Defects

NIAAA-sponsored studies have shown that chronic use of alcohol impairs the body’s immune system, possibly setting the stage for respiratory and liver infections as well as AIDS (Roselle 1992; Rosman 1992; Kruger and Jerrells 1992).

## Prevention of Alcohol-Related Problems

During recent years investigators have placed increasing priority on researching the prevention of alcohol-related problems. Until recently, prevention efforts were essentially limited to educational programs to inform people of the dangers of alcohol abuse and alcoholism. During the past 10 to 15 years, there has been an explosion of interest in complementing educational approaches with strategies aimed at altering the social, legal, and economic context in which drinking occurs (Holder and Wallack 1986).

### Effects of Reduced Access to Alcohol

A common approach to preventing alcohol problems is to decrease the availability of alcohol through manipulation of price or restrictions on the sale of alcoholic beverages. Some scientists have argued that availability restrictions affect only light and moderate drinkers, because alcoholics will accept any inconvenience or expense to satisfy their addiction. Ledermann, a French medical demographer, challenged this assumption, suggesting for the first time that heavy drinking is sensitive to changes in the same supply-and-demand factors that govern consumer behavior in general. NIAAA-sponsored research has confirmed that reduced access to alcoholic beverages can be associated with reductions in the medical and social consequences of heavy drinking, especially among youth (Chaloupka 1992; Grossman et al. 1987; Coate and Grossman 1988). Based on computer modeling studies, Grossman and colleagues (1991) predicted that increases in alcoholic beverage taxes might be an effective means of reducing alcohol-involved driving and related traffic fatalities. Researchers have not yet determined whether manipulation of price would have an equal effect on different beverage types and on different subpopulations of drinkers.

### Integrating Targeted and Broad-Based Prevention Approaches

Once it was assumed that most alcohol-related problems result from heavy drinking by alcoholics, who were therefore viewed as the appropriate target for preventive and treatment efforts. However, many people who experience alcohol-related problems are light to moderate drinkers who are not addicted to alcohol. Although these people are at far less risk individually than are heavy drinkers, they form a much greater proportion of the population (Kreitman 1986). Indeed, even a single episode of alcohol misuse can have adverse consequences—including death, as in the case of drinking and driving. Researchers are therefore seeking ways to complement targeted interventions that focus on high-risk populations with strategies aimed at the entire population of drinkers.

## Research Helps Influence Public Policy

### Prevention of Traffic Fatalities

NIAAA research provided the scientific basis for the Federal Uniform Drinking Age Act of 1984, which specified that Federal highway funds would be withheld from any State that did not set 21 as the minimum legal drinking age (MLDA) for purchase or public possession of all alcoholic beverages. Subsequent studies have reported that raising the MLDA from 18 to 21 years of age has dramatically reduced alcohol-related driving deaths, especially among persons ages 16 to 20 (NIAAA 1993*c*; O’Malley and Wagenaar 1991; Hingson and Howland 1989).

### Liver Transplantation for Alcoholic Liver Disease

Scientific data also were conclusive in shaping policy concerning liver transplants for persons with alcoholic liver disease. Liver transplantation is the only effective treatment for patients with end-stage cirrhosis. However, many clinicians believed that alcoholic liver recipients would relapse into drinking, thereby destroying their new liver. Data showed that the survival of alcoholic cirrhosis patients after transplant was no worse than that for patients with nonalcoholic liver disease (Starzl et al. 1988). In addition, the rate of relapse to drinking among transplant recipients was low.^2^ Based on these data, medicare rules were revised to include alcoholic liver disease as a condition for which liver transplantation is considered accepted medical practice.

### Fetal Alcohol Syndrome

Beginning in 1989 Federal law has required that all alcoholic beverage containers sold or distributed in the United States bear warning labels. One such label addresses pregnant women as follows: “According to the Surgeon General, women should not drink alcoholic beverages during pregnancy because of the risk of birth defects.” Meanwhile, for the past decade, NIAAA has sponsored a nationwide effort to alert clinicians and expectant mothers of the risks of maternal drinking to the fetus.

## Treatment Research

Alcoholism treatment methods traditionally had been developed on the basis of clinical experience and intuition, with little rigorous validation of their effectiveness (Woody et al. 1991). The rise of health care costs has accelerated the need for research to document which treatments succeed, for whom, and how. Over the past 10 years, alcoholism treatment research has employed modern standards of outcome evaluation, such as the use of control groups for comparison purposes, random assignment of subjects to treatments, multiple and objective measures of treatment effects, adequate followup to confirm results, and appropriate statistical analysis (Fuller 1990). Results of these studies will permit researchers to improve treatments and will ensure that clinicians can provide the best available treatment to their patients.

An important development in treatment research is a new emphasis on patient-treatment matching. Because no single treatment approach is effective for all people with alcohol problems, NIAAA is sponsoring Project MATCH to develop practical guidelines for assigning patients to appropriate treatments based on relevant patient characteristics. Involving 1,700 patients at 9 sites, Project MATCH is the largest and most complex trial of patient-treatment matching and treatment effectiveness ever undertaken. Although the first 5 years of the program concluded in the fall of 1994, researchers are continuing to analyze the extensive data collected (Mattson 1994).

Modern research standards also have been applied to determine the effectiveness of disulfiram (Antabuse^®^), a medication used since 1949 to discourage drinking without convincing evidence of efficacy. In a large, multisite, double-blind controlled clinical trial conducted by the Veterans Administration (Fuller et al. 1986), researchers found that disulfiram itself was not associated with increased total abstinence. However, despite lack of compliance among many patients, subjects who remained in the study and continued to take their disulfiram exhibited a significantly decreased number of drinking days.

Controlled clinical trials supported by NIAAA have led to the Food and Drug Administration’s recent approval of the use of naltrexone to assist alcoholics in treatment to maintain abstinence (O’Malley et al. 1992; Volpicelli et al. 1992). A product of neuroscience research, naltrexone is the first new medication in 45 years approved to help maintain sobriety after detoxification from alcohol.

The long-term goal of NIAAA-sponsored clinical research is to develop a knowledge base for improving the efficiency and effectiveness of treatment delivery for alcohol-related problems. NIAAA currently funds multidisciplinary studies on the impact of organization, financing, management, and delivery of alcohol-related health care on service accessibility, quality, utilization, cost, and outcome. Ultimately these studies should ensure that the public is offered the highest quality of alcohol-related prevention and treatment services at the lowest possible cost.

## Where Are We Now?

The past 25 years of research have both diversified and consolidated our knowledge of alcohol problems. On the molecular scale, it appears that the diversity of alcohol’s addictive and medical effects can be attributed to its interference with a small number of key molecular processes common to all cells of the body. As basic research expands our knowledge base, a consistent pattern appears to be emerging that ultimately will help guide improved prevention and treatment efforts.

On both individual and societal levels, however, alcohol-related problems display a heterogeneity that challenges historical notions of alcoholism as a one-dimensional problem. The causes and manifestations of alcoholism differ from one person to another, sometimes markedly. Multiple genes may each make contributions toward the disorder, and some researchers have suggested that certain families may have their own unique mixture of genes responsible for alcoholism susceptibility (Plomin 1990). In addition, the relative contributions of genetic and environmental factors to the development of alcoholism differ in different patients. Genetic influences appear to predominate for some people, whereas environmental influences appear to predominate for others (Pickens and Svikis 1991). These interactions appear to fluctuate over time, and risk for alcoholism or problem drinking may emerge at any age.

## Where Are We Going?

The potential for synergism among the various scientific disciplines in alcohol research is greater than ever since NIAAA has become part of the National Institutes of Health. As we approach the beginning of a new century, research efforts will concentrate on the following:

- Determining which aspects of the vulnerability to alcoholism are inherited. Linking specific traits with specific genes or groups of genes may support innovative ways to interfere with the development of alcoholism.
- Determining how genetic and non-genetic factors interact in the development of alcoholism. The ability of some nerve cells to undergo long-term adaptive changes in response to environmental stimuli suggests that nature and nurture cannot be considered separately in the development of alcoholism.
- Increasing efforts to address the special treatment needs of traditionally underserved populations. For example, NIAAA supports the largest and most intensive study of women’s drinking habits over time ever conducted in the United States as well as research to develop effective treatments aimed at ethnic and racial minorities, older individuals, and youth.
- Developing new treatment methods. For example, NIAAA researchers are searching for medications that act to impede the progression of alcoholism and lessen the risk of relapse in recovering alcoholics. In the near future, this research may be aided by computer-assisted molecular technology for predicting the therapeutic effects of potential medications.

Finally, alcohol research will help remove the stigma associated with alcoholism as it provides hope for the millions of people affected by this disease, their families, and society.

## Footnotes

1 Receptors function by recognizing and responding to specific molecules, called neurotransmitters, released by nerve cells.

2 Recent research suggests that outcome is enhanced by appropriate patient selection procedures and continuous followup care (reviewed in Beresford 1994).
